# Acceptability of Guided Symptom Entry and Asynchronous Clinical Communication Software Among Primary Care Staff: Qualitative Study

**DOI:** 10.2196/59620

**Published:** 2025-07-16

**Authors:** Riina Raudne, Taavi Tillmann

**Affiliations:** 1 Institute of Family Medicine and Public Health, University of Tartu, Ravila 19, Tartu, 50411, Estonia, 372 53426963

**Keywords:** health information exchange, telemedicine, symptom entry, health portal, online consultations, cognitive load, user experience, E-Visits, asynchronous care, primary care.

## Abstract

**Background:**

Patients often communicate with primary care centers remotely (eg, by telephone or email) before seeking in-person care. A comparatively novel addition might be patient-facing symptom entry websites, where subsequent questions are automatically guided by previous responses. However, the acceptability of such systems to health care staff remains unclear, particularly in terms of what features staff perceive as useful.

**Objective:**

This study aimed to investigate a patient-facing algorithm-guided symptom-entry software (developed by Certific OÜ, Estonia), which also supports subsequent asynchronous communication, for its acceptability and perceived utility to primary health care providers.

**Methods:**

In-depth and open-ended interviews were conducted in 8 primary care centers in Estonia, including 8 nurses and 6 doctors, 3‐6 months after the implementation of a novel patient-facing website. Transcripts were coded inductively, using grounded theory and phenomenological approaches to uncover themes most salient to providers. Two family doctors provided feedback on the final analysis.

**Results:**

Staff perceived unstructured communication (via email and phone calls) as a burden that increased their cognitive load. Sometimes, this arises out of the perceived mismatch between needing to identify and document critical symptom information and being unable to standardize the supply of such information, due to a heterogeneous and unpredictable communication processes whose duration, quality, and risk of miscommunication are hard to predict and control. All interviewees expressed the desire that more patients initiate their remote query via the algorithm-guided symptom-entry software. The software was reported to satisfy perceived feature needs for patient verification, privacy and data security, editable plain-language symptom summaries of symptoms, and integration with prewritten response templates (particularly for staff who were nonnative speakers). Safety of the new software was perceived as high, on account of integration alongside traditional telephone requests. Staff reported the challenge that great effort was needed to persuade patients to use the website. Among perceived challenges, some providers reported difficulty in onboarding patients, digital literacy gaps, and limited time savings. While previous research has criticized poorly designed multiple-choice systems, our findings suggest that an appropriately designed and personalized multiple-choice system can be preferable to health care staff, as they may lower cognitive demands and enhance well-being.

**Conclusions:**

Interviewed primary health care staff felt that this symptom entry software was acceptable and desirable. They valued a perceived reduction in cognitive demands. This holds promise for increasing staff well-being and increasing efficiency, which needs to be quantified in future studies.

## Introduction

### Literature Review

Historically, most primary care patients discussed most of their symptoms with a health care provider face-to-face. Usually, this was preceded by registering new patients using paper documentation, and appointments were scheduled by short phone calls with receptionists. Various information and communication technologies have disrupted this model of care, particularly after the pandemic. Initially, these focused on replacing synchronous in-person care with synchronous online care (eg by switching to a video, phone, or instant messaging communication route) where the traditional concept of a time-limited “medical appointment” was maintained [1,2]. More recently, asynchronous technologies are emerging to complement, and in some instances, replace aspects of synchronous care. Although its terminology continues to evolve, typically these technologies span one or many domains of the following 5 chronological processes: (1) patient intake technology allows new patients to register and identify themselves online without an in-person visit; (2) symptom entry websites typically have a one-size-fits all structure and do not adapt in the light of previous responses; (3) symptom data analytics include self-triage and the provision of automated medical advice; (4) symptom data transmission software allows patient-entered data to be read and actioned by clinicians at an asynchronous timepoint; and (5) patient-clinician communication platforms enable bidirectional message exchange in either a synchronous or asynchronous manner [3].

These 5 domains could be seen as modular, whereby health care providers may use multiple IT providers whose functions may overlap with one another, and some of the 5 domains may not be provided. Sometimes, the integration of many of these domains may allow simpler and lower-risk queries to be handled and closed without requiring an in-person visit, and this is sometimes referred to as electronic visits or (e-visits) [4].

Academic study of principally asynchronous clinical care technologies may help inform applied questions of if, where, when and how to adopt certain technologies and how these technologies may be improved [1,5]. Patient satisfaction with asynchronous care appears high [6-8]; however, clinician acceptability has been less studied. The few quantitative studies to date have found mixed findings, with some suggesting that clinicians may prefer asynchronous to synchronous models of communication [9], while others reporting low levels of acceptability [10-13], potentially due to increased cognitive exhaustion from taking in information in new formats [14-16]. One theoretical model suggested that online consultation technologies could ease physician burdens if they do not spur higher demand from patients and can resolve half of patients’ queries without face-to-face follow-up [17]. However, most platforms have not met these criteria in evaluations, thereby risking exacerbating physician workloads. What else clinicians want from such software, and how the technology can deliver this, remains understudied and unclear [6]. Previous research has identified some features that have facilitated greater acceptability by clinicians, such as direct integration with electronic health record (EHRs), accurate patient identification, trust in privacy and security, and quasi-automated triage [7,8,11], more complex workflow such as initial nurse review before escalating to physicians, and free text options [1,17].

### Certific’s Guided Symptom-Entry and Asynchronous Communication Software

Patients and clinicians access a secure website using any internet-enabled device (incl. smartphones and PCs), without needing new software or hardware. A multifactor authentication process is designed to be tailored for each country. In Estonia (the only country where Certific currently operates), all residents are identified by a 11-digit personal identification code, which users operationalize for digital identification by one of 3 hardware options: Smart-ID is a phone app for smartphones that is initially activated in-person at a police station or bank branch; Mobile-ID is an SMS text messaging-based verification service activated by telecommunications operators, making it suitable for older mobile phones; and the combination of a PC or mac with a USB-card reader, plus a credit-card sized national ID-card*,* allows for digital identification for those without mobiles (including at public PCs).

Patients select their presenting complaint from a list of available options (eg, headache). A short adaptive questionnaire covers related symptoms (eg neck stiffness), onset, and a text box. The pathways are tailored to dynamically adapt in light of the severity of the data entered, with intent of reducing the need for follow-up queries and supporting faster decision-making. As optional features, patients can submit a health complaint on behalf of a relative or a next of kin, and upload images.

The software does not provide triage or urgency categorization. It lists the presenting complaint in the query title, thereby facilitating clinicians when performing triage as they scan lists of query titles. After opening a query, clinicians view a structured, plain-language summary of the presenting complaint. In case of a mismatch between the languages used by patients and clinicians, clinicians may opt to view a machine translation version (provided by DeepL). They can edit this summary before manually copying and pasting it into an external EHR system. Depending on the complexity of the query, clinicians can close it outright, send an instant (or delayed) message (or SMS text message) to the patient, or invite them for an appointment. Many of these can be streamlined by using predefined or user-generated templates.

Once a clinician has responded, patients receive a short email notification directing them to view the detailed message on the secure platform. Patients may also respond to the clinician’s message (if permitted by the clinician).

The software has been available in Estonia since the beginning of 2023, with 69 primary care centers (out of the country’s 400, giving about one-fifth market share) subscribing to its services at the end of 2023, when this study was done. For further background about the types of patients who were using the system during this time, the IT service provider (Certific OÜ) has described their demographics and presenting complaints in Multimedia Appendix 1. This appendix also lists further technical details and screenshots that illustrate the above text.

For additional context, many Estonian primary care practices have permitted unverified individuals to email their practice with medical queries since the late 2000s. In 2014, the Estonian National Health Insurance Fund (which is similar to the National Health Service in the United Kingdom) formally recognized e-consultations (when general practitioners ask advice from other specialists using remote asynchronous communication like email) as an official and billable health care activity. Thus the culture of asking for medical advice over email has been long widespread in the Estonian health care system.

This study investigates the perceived experiences, utility, and acceptability reported by primary care clinicians shortly after the adoption Certific’s software, which enables algorithm-guided symptom-entry for patients, and its asynchronous medical review and communication by clinicians.

## Methods

### The Research Team

The core research team consisted of the 2 coauthors of this article. We had quarterly meetings with 2 staff working at the IT provider (Certific), Andres Lasn and Helena Volmer, whose input helped inform the study design, interview guide, and sampling approach. All interviews were conducted, and the qualitative analysis was led by one academic experienced in qualitative research (first author RR) with a public health background, either in person or via video conferencing. The researcher did not have previous expert knowledge on web-based consultation software, but was experienced with health services, and generic software development, so she conducted interviews as someone professionally familiar with the health system. The researcher was employed exclusively at the affiliated university to work on this project; there was no prior or subsequent professional or personal relationship with Certific. The second researcher (TT) is an academic clinician experienced in public health interventions, including the design, implementation, and evaluation of digital public health tools. He has previously not worked with web-based consultation software for symptomatic patients. He provided the theoretical context for the study and helped interpret findings in light of existing digital health literature.

Certific assisted with drawing up the purposive sample and sending out introductory recruitment emails. The interviewer had no prior relationship with any of the interviewees. The participants were told by Certific that a researcher from the University of Tartu is seeking feedback on provider experiences with Certific, as part of a qualitative study. Both researchers were motivated to work on this project out of interest in innovation in health services and an opportunity to learn and contribute to health technology development. They are otherwise not involved with, and have no competing interests in, this or similar IT solutions that address the health care needs of symptomatic patients in primary care.

### Study Design

This qualitative study assessed the perceived feasibility, utility and acceptability among primary health care staff in Estonia during the first months after implementing Certific’s communication technology. Its open-ended design sought primary care providers’ rich descriptions and own explanations, perceptions, mental models and framings. Rather than using and potentially departing from established theories, frameworks and metrics of what might potentially makes one software acceptable to primary care staff, we asked participants to tell us in their own words what they perceive remote consulting to mean, what are its aims, channels, and which tools allow them to best achieve what they are trying to do.

Grounded theory and phenomenological approaches were employed in all phases of study design to prioritize providers' own descriptions of challenges in their practice and experiences (phenomenological aim) with remote consulting, including Certific. Emergent themes were also systematically analyzed (grounded theory aim). This analytic approach was preferred to more predefined evaluation frameworks, because one of the aims in our funding proposal (which we had to adhere to in delivering the research) was to use this independent formative evaluation to quickly contribute to software development, by feeding back important user needs to the software developer. Accordingly, open-ended interviews with relatively few questions and only one researcher conducting the interviews were chosen as a method of data collection. As compared to focus groups or interviews with several researchers, conversations with solo researchers have limitations (see Discussion); however, they can also increase privacy and trust, potentially leading to more disclosive conversations.

### Recruitment

Participants were purposively sampled from among Certific’s recently onboarded clients. In a relatively small and homogeneous country like Estonia (with just 1.3 million inhabitants), we sought maximum variety in practice location, size, and demographics. Recruitment and interviews took place from May to September 2023, involving both native-Estonian and native Russian-speaking professionals proficient in Estonian. Invitations were sent via email to primary care physicians and nurses, with 6 physicians and 8 nurses consenting to participate. One invited recipient declined, due to increased workload owing to the summer vacations. Altogether, 14 face-to-face interviews were conducted either in person or via video conferencing. There was one repeat interview with a doctor at the end of the initial analysis to check our interpretation of core findings. One interview involved two nurses who wished to be interviewed together.

### Data Collection

The interviews, which were audio-recorded and transcribed, explored participants' views on the current realities and changing nature of their roles and their practices, especially in the context of the increasing role of remote consultations. The openly framed questions of the interview guide (Textbox 1) sought provider perceptions of strengths and weaknesses of using different remote communication tools such as the telephone, email, and other online consultation platforms, if applicable, in the context of what providers sought to achieve. Finally, we enquired about the specific experiences with Certific.

Textbox 1. **Interview guide used in the interviews**

Introduction:

- Please tell us a little bit about your role and your practice. How is it changing?
- What are the current main challenges?

On the rise of remote consulting:

- How is remote care organized in your practice? What channels do you use for patient communication?
- What are the benefits and drawbacks of each communication channel?

- Phone
- Email
- Online consultation software

On Certific:

- Can you try to summarize, with very short and simple words what Certific’s software is and does?
- How and why did your practice decide to adopt this software?
- What has been your experience with it so far?
- What are its results and impacts, for example, has it changed workflow?
- What are the upsides and downsides of Certific’s software when compared to other options?
- Can you walk me through exactly what happens when a patient reaches out to you via Certific?
- What other tools do you have to complete one patient inquiry?
- What is missing at the moment from this software?

### Data Analysis

Interviews were transcribed verbatim and coded by the same researcher (RR) who conducted the interviews using Atlas.ti software. Analysis involved a systematic reflection on the collected data during the interview and coding phases. Some themes were identified beforehand and resulted from the interview guide (such as the benefits and drawbacks of phone, email, and Certific software in solving patient’s queries). Other themes and surprising subthemes emerged during the analysis, such as perceived increases in workload arising from greater patients' (remote) access to care; overuse of medical services; desire for greater gatekeeping to prevent undue use; variable digital literacy among patients; unpredictable quality of email queries; and descriptions of the mental load while staff try to identify and categorize key diagnostic features.

Emergent themes and findings were discussed throughout, during meetings with the study team and with Certific to provide feedback. A version of the analysis was also presented to 2 family doctors, one of whom had also been interviewed, for sense-checking and feedback. When drafting this article, we followed the "Consolidated criteria for reporting qualitative research (COREQ): a 32-item checklist for interviews and focus groups" which is provided in Checklist 1.

### Ethical Considerations

Formal ethics approval was not required according to Estonian regulations and the Oviedo Convention [18], as this study involved voluntary interviews with health care professionals about their professional roles, posed minimal risk, and collected no sensitive personal or patient data. All participants gave informed consent for recording, transcription, and pseudonymized publication of quotations. Participants were not compensated for their time (as this took place during usual work hours). This approach aligns with the Estonian Code of Conduct for Research Integrity [19], which stipulates that researchers must obtain ethics approval when required by law or institutional rules, but allows for researcher discretion in low-risk studies involving professionals and nonsensitive data. In addition, the study complies with the principles outlined in the Oviedo Convention (Convention on Human Rights and Biomedicine), which Estonia ratified in 2002. The Convention emphasizes the protection of human rights in the biomedical field and allows for national discretion in ethical oversight of research not involving medical interventions or sensitive personal data [20].

### Role of the Funder

The study was funded by the Estonian government’s Applied Research Program. Specifically, this qualitative study was one part of a larger grant made available to the software developer, to provide an external and independent academic assessment.

## Results

### Overview

We interviewed 14 primary care providers (8 nurses and 6 doctors) between June and September 2023. Certific’s software had been in use for 3‐6 months before our interview. The number of patients per center ranged from 1500 to 10,000, with staff numbers ranging from 2 to 16. All centers used telephone, website, Gmail-based email, and Certific’s software for remote communication; 3 clinicians had also used other remote consultation software. During the initial open-ended interviews, some new themes (with example types that turned into codes) emerged such as provider cognitive load and the communication patterns of patients. Time and follow-up attention was afforded for these themes to be fully elaborated in subsequent interviews. Saturation was noted in all reported themes.

The following is a summary of selected themes that emerged in data analysis. Examples of coding trees are presented in Multimedia Appendix 2.

### How Remote Consultations Have Changed Doctor-Patient Communication and Demand

Interviewees described significant changes in patient communication during the recent years Multimedia Appendix 3. While patient uptake of remote consultations varied, COVID-19 had made remote consulting the new default. Several practices had stopped and never reintroduced walk-ins without previous appointments.

### Remote Consulting Using Email

Face-to-face contact enables providers to quickly carry out multisensory examinations of patients’ signs and symptoms. Their omission over email revealed a significant variability in patients’ ability to communicate their symptoms in writing. This creates additional work for staff, who have to identify, categorize, identify uncertainty, and sometimes narrow this by explicitly asking about critical diagnostic features from lengthy verbose texts or vague, unsigned and sometimes impolite one-line questions that echoe chat culture. Often, patients’ emails were incomplete, missing critical administrative details or symptoms necessary to complete a task.

Well, this patient just wants to tell me all their concerns in one big sitting, but I have no use for all this excess information… this kind of massive letter I can’t even copy and I have to spend considerable time in distilling it…

[GP 4]

The aim of good software might be that such patients' can't write all those endless lengthy emails

[GP 3]

Staff are required to record a summary of each patient interaction in an EHR system. Staff said that it had become a significant challenge during the remote era to interpret patients’ written queries and asynchronously follow up for isolated critical details across multiple channels (such as a long email, followed by a short telephone call) to quickly compile a sufficient summary of the patient’s symptoms.

In general, interviewees were not concerned about remote access for older and less digitally literate patient groups, having witnessed their adaptation to email over the past decade. However, providers were more concerned about the ways in which email in particular appeared almost too convenient of a method for reaching out to doctors and they perceived an increased demand they considered sometimes as frivolous and unnecessary:

There are some people who are completely helpless. One week they call and say their child has a fever. I ask if they’ve taken their temperature and given paracetamol. They haven’t. So I tell them to give some paracetamol, use steam, etc. Our nurse explains everything in detail, which takes about 10 minutes. They then want a sick note. The child recovers. Three weeks later, the same child develops the same symptoms, and we go through exactly the same steps once again. The nurse repeats the exact same advice, asks if they took paracetamol. They have not. I understand if you call once a year, but calling twice a month to be told to take paracetamol is ridiculous. This could easily be handled by a chatbot.

[GP 4]

The downside of email is that someone can have a thought at 1 AM, and they pour it all into an email. Then in the morning, I read it all and think, 'Wow, someone was in a bad mood last night.' But with remote consultation where you have to first log in and jump through hoops, people don’t bother as much. Emails are just too accessible; everything comes through. It’s too easy to send messages, and I already know some people here who vent a lot in their emails. I know I don’t have to respond to everything, and sometimes I think it’s nice that people can vent, but it’s simply overwhelming.

[Nurse 8]

### Remote Consulting Using Phone

Providers described synchronous phone-based communication to have both advantages and disadvantages. First, hearing vocal cues brought back additional multisensory signs that staff have been trained for, and increased confidence in their diagnostic abilities.

Second, synchronous interactions allow perceived information gaps to be completed, follow-up appointments to be arranged, and understanding to be checked quickly.

The option for patients to phone the practice was perceived as the most accessible alternative to in-person appointments. As practices were contemplating options such as restricting email access and walk-ins, knowing that their practice offers telephone access as a back-up to those in need provided a relief for concerns about potential unequal access.

As drawbacks, telephone calls required simultaneously focusing on listening and typing for documentation, the potential for information to get lost in this transfer from speech to text, and disruptions into other ongoing tasks that call-backs caused. Participants noted the stress of handling emotional or upset patients over the phone, where they had to simultaneously listen, document, diagnose, and search for helpful information and communication routes while maintaining adequate awareness and control over their own feelings.

The combination of email and phone created a time-pressed, stressful, and fragmented flow of communication, particularly for nurses who typically were frontline responders.

Especially during the flu season, several providers reported coming to work on Monday mornings and finding an overflow of email queries, around 50 to handle in one day. Larger practices had established clearer workloads, such as “never letting one person respond to remote queries for more than 4 hours at a time.” Smaller practices did not have such luxury of choice, and nurses expressed a sentiment of despondent acceptance.

### First and Subsequent Impressions

*At the beginning I thought ‘Gosh, not another login, not another window to look at. But in hindsight I was mistaken. Now I really like this program.*

[Nurse 6]

### Data Security and Identity Verification

In most cases, Certific was first adopted for its data security and privacy capabilities, as Gmail was deemed insufficient for meeting legal requirements.

*Frankly, Gmail is still a little bit more convenient and familiar, but we know that it’s not secure. That’s why we adopted Certific. We had to make a choice as something had to replace Gmail.*

[GP 1]

The use of national ID for login streamlined patient identification, reducing the burden of confirming identities from ambiguous emails.

Sometimes I get an e-mail from, say ‘abcde999@hot.ee’ (a made-up email address) and then I have to begin from writing back to them and asking what their name is and whether they are really our patient in the first place.

[GP 4]

On the other hand, the identification requirement on the patient side was also deemed by the providers to constitute a suitable barrier to appropriately help reduce excess demand.

The ease of writing an email leads people to send messages impulsively—an idea pops into their head, and they immediately write to us. Certific requires a bit more effort from them. Now, sometimes we get people writing to us with just one sentence, without even a greeting or closing. Communicating via Gmail is too convenient; its liberties are frankly sometimes abused. A single thought comes to the patient’s mind, and they write to the doctor straight away.

[GP 2]

### Structure and Clarity in Communication

Participants reported that Certific’s software brought more structure and clarity to patient communications. The software’s guided questions on symptoms was perceived to provide in most cases a comprehensive summary for subsequent triage, and to also educate patients about the information that clinicians might need:

*What I like is that patients approach us through guided questions. This forces the person to give a little thought to their request, those guiding questions are very good. On the phone or via email they will just say the first things that comes to their mind.*

[Nurse 2]

In addition, participants noted that the requirement to log in provided a barrier to excessive convenience.

*It kind of disciplines the patient. Otherwise, we have to ask over three or four emails where to direct their attention. But now they can directly just talk to the machine…*

[Nurse 4]

### Documentation Ease and Standard Response Templates

Interviewees appreciated Certific’s structured and plain language summaries, which filtered out less relevant details. This reduces the effort needed to synthesize a summary and it was perceived to free up time to focus on speaking with patients. The editable templates and links to guidelines reduced workload, particularly during high-volume periods.

*I think nurses have to answer a lot of questions from people with colds and coughs. Now that they have standard template answers, I think things have improved for them.*

[GP 6]

### Reduction of Emotional Burden

Patient queries through Certific’s software provided relatively complete case facts, minimizing the need for follow-up questions and allowing pauses before responding to patients. This reportedly mitigated stress from continuous communication with unwell patients and occasional encounters with rudeness.

### Time-Saving Perceptions

When asked whether the use of Certific OC platform saves time, most providers were not sure. In their perception, Certific’s software had nonetheless improved the ease, structure, and clarity of case management, contributing to a more efficient workflow.

I cannot say that I am spending less (time solving a patient’s case) than on the phone or on e-mail, I think it’ll end up around the same amount of time. It’s just that… now it’s very convenient that when I respond, I click-click-click on prefilled fields… I cannot entirely automate it, I still have to focus and think about it, and add little notes.

[Nurse 7]

### Challenges With Implementation and Patient Reception

Despite the improvements, remote consultation on a platform such as Certific’s could not, according to participants, replace the immediacy of face-to-face consultations and telephone, which participants considered best for lowering diagnostic risks by getting extra sensory input about a patient’s condition through the voice, body language, and general vitality.

Concerns were raised by some staff about the patient digital literacy required for using the software (eg logging in), especially among older patients in smaller towns. However, other staff elaborated how phone and email were still sufficient options for these patients, and they did not express accessibility concerns. Some patients reportedly opposed the mandatory requirement of accepting “cookies” on Certific’s website, leading them to opt-out of the service.

### Patient Onboarding

Informing patients about adopting Certific’s software was described as slow and complex, with centers lacking the resources for marketing and onboarding. During data collection, patients mainly learned about this communication route reactively, when approaching their primary physician often in person, as few proactive and systematic communication attempts were made by centres due to incomplete email lists.

Furthermore, a temporary technical inability to transfer all patient contacts to Certific at the time of conducting the interviews, requiring manual contact entry, had temporarily slowed workflows and reduced motivation to invest time in the platform. This problem has since been solved, alongside a new onboarding process that allows SMS text messaging notification campaigns to all registered patients; email footers, prerecorded phone messages, and website links.

## Discussion

### Principal Findings

In this qualitative study, we found that primary care doctors and nurses in Estonia supported novel technological solutions to the historical problem of overwhelming unstructured written information when patients emailed practices with free text that often led to long-winded need for clarifications. The most useful feature that persuaded clinicians to consider trying the software in the first place was its ability to deliver a high degree of security and privacy against the risk of sensitive medical information being leaked via insecure means, such as Gmail servers residing around the world. Though participants described greater ease of managing patient cases using Certific, this did not translate into provider perceptions of saved time. Rather, the main perceived benefits after having adopting the platform after a few months came from the number of cognitively challenging tasks that had been automated (eg summarizing patient symptom descriptions, providing pre-written response templates and links to treatment guidelines, reducing the need to formulate replies for frequent problems from scratch).

### Comparison With Previous Work

Previous research has identified technological features that facilitate greater acceptability by clinicians. Some of these features also emerged in our study, adding consistency to their transferability, such as direct data transfer into the electronic health record and other integrations; initial review by nurse before escalating to physician [21]; accurate identification of patients, privacy, security and confidentiality [20]; and quasi-automated triage including self-care and in-person options [1]. However, while previous studies suggested that free-text entry is superior for capturing patient queries [1,22], our findings contradict this. Clinicians in our study felt overwhelmed by free text and preferred the plain language summaries generated from multiple-choice symptom-entry systems, which minimized the need for follow-up.

The design of Certific’s multiple choice system, which guides patients through thematic and adaptive symptom-gathering pathways and ensures all guideline-relevant questions are asked, seemingly improves the signal-to-noise ratio of the output summary [10,14,23]. A related consideration is how appropriately the remote system is embedded into other modalities of care. Excessively strict gatekeeping of in-person care can lead to patients manipulating their symptoms to get appointments [7,10], but this was not an issue in our study as in-person appointments were usually available within a week.

### Suggestions for Future Research

First, our findings suggest that a well-designed, guided and highly personalized multiple choice symptom entry system may for staff lower improve cognitive demands, and increase workplace satisfaction and well-being. Future studies could evaluate this quantitatively, using questionnaires or absence data, ideally using a randomized design. Second, there remains considerable uncertainty whether this may increase or decrease total work time. As it is difficult to technologically measure how many seconds a staff member uses one or 2 IT systems (especially if these are being used in parallel) while concurrently responding to email, telephone calls, or in-person conversations, a traditional design using an observer with a stopwatch may help shed light on this question. Third, consistent with previous studies, we observed patients who used the system had more younger and female patients. This risks marginalizing older and male patients. Service developers could consider explicitly involving them, to design services around this demographic. Other dimensions of equity (including patients’ education, income, and ethnicity, etc) should also be monitored and services developed in an inequality-reducing manner, where possible.

### Limitations

This study has several constraints, which may have lowered the trustworthiness and credibility of our findings. First, the limited sample size, stemming from a small number of potentially “early adopter” primary care practices who had employed this software at this early phase, in one country, could limit broader transferability. More specifically, we conducted comparatively few interviews with those health care staff such as those who were working in rural areas, male staff members, and doctors. Second, the study did not investigate how acceptable the software was to patients. Third, centres participating in the study had invested effort and finances into Certific, potentially introducing plan continuation bias, the psychological need to defend a previous decision. Fourth, a single researcher both collected the data and analyzed it. Despite regular team discussions, the single-researcher approach (while offering certain advantages) may have increased the risk of personal perceptions and biases influencing data collection and analysis. For example, this interviewer’s background and interest in patient journeys and user journey may have influenced the choice of themes that she found most relevant (both in terms of deciding which clues to further elaborate during the interview, and which themes were pursued during the analysis (refer Multimedia Appendix 2 - coding tree). Furthermore, empathy towards health care providers and their communication struggles grew over the course of the interviews, possibly impacting the analysis by siding with the health care staff members’ perspective. However, these limitations were partly offset by all interviewees having no prior contact with the interviewer, and the interviewer being broadly trained and experienced in understanding and managing such biases as best as possible.

### Conclusions

Primary health care staff in Estonia felt that Certific’s guided symptom entry software, which facilitates subsequent asynchronous clinical communication, was acceptable and desirable. When compared to the conventional alternative of emails and telephone calls, staff initially felt an interest in trialing the software due to its superior security and privacy features. After a few months of use, they reported a perceived reduction in cognitive demands when compared to email and telephone routes. This holds promise for increasing staff well-being, which could be assessed in future studies. Staff did not explicitly perceive the software to lead to time savings, which also be assessed in future studies.

## Supplementary material

10.2196/59620 Multimedia Appendix 1 Technical details of Certific's system (Design, Ux, UI and workflow), plus presenting complaints made by typical patients.

10.2196/59620 Multimedia Appendix 2 Coding tree used in the analysis.

10.2196/59620 Multimedia Appendix 3 Description of the sample used in recruitment of interviewees.

10.2196/59620 Checklist 1 Consolidated criteria for reporting qualitative research (COREQ): a 32-item checklist for interviews and focus groups - completed by the study authors.

## Abbreviations

COREQ: Consolidated criteria for reporting qualitative research
EHR: electronic health record
