# Supplementary material for: Acceptability of Guided Symptom Entry and Asynchronous Clinical Communication Software Among Primary Care Staff: Qualitative Study
Source: JMIR Form Res. 2025 Jul 16;9:e59620. doi: 10.2196/59620 (PMC12286587; doi:10.2196/59620)
Supplement: Multimedia Appendix 1 [file formative-v9-e59620-s001.docx]

**Multimedia Appendix 1:**

**Technical details of Certific's system (Design, Ux/UI and workflow)**

**plus presenting complaints made by typical patients**

**History of this software in Estonia**

Certific is a doctor-patient communication platform designed to facilitate the structured exchange of patient health information. It aims to streamline clinical workflows and reduce the administrative tasks associated with managing patient data.

Since its launch in Estonia on January 1, 2022, and as of November 24, 2024, it has become available to 140 clinics, 1,020 primary care clinicians (of whom 59% are nurses and 41% are doctors), and 430,200 patients.

At the time of data analysis for this study, on September 13, 2023, the platform was in use by 42 clinics. At this time, IT costs were borne exclusively by the primary care centre. Shortly after the end of data collection in this study (01.2024), these IT-costs have been co-financed by the Estonian Health Insurance Fund, which may have contributed to greater subsequent adoption. As part of securing this funding, the software successfully underwent an audit conducted by the Estonian Health Insurance Fund to verify its compliance with national and European data security and privacy standards.

**Design Principles and Workflow**

Certific’s platform uses condition-based, dynamic questionnaires that guide patients through reporting their symptoms. The system prioritizes structured data collection using multiple-choice options while allowing some free-text inputs when needed. This approach minimizes the need for follow-up questions by ensuring that the initial data submitted by the patient is both concise and clinically relevant. The system's design emphasizes reducing the cognitive burden on clinicians, as they receive more structured information at the onset of the consultation process. Certific provides clinics with a pre-visit anamnesis that clinicians can review and transfer into the Electronic Health Record (EHR) with minimal manual input. The platform includes a feature that allows clinicians to manage the flow of communication with patients. Clinicians can determine whether to allow patients to respond immediately, delay the response for a set time (e.g., after three days), or close the conversation entirely once the case is resolved. This feature is designed to limit unnecessary follow-up exchanges that might otherwise occur in more open communication systems such as email.


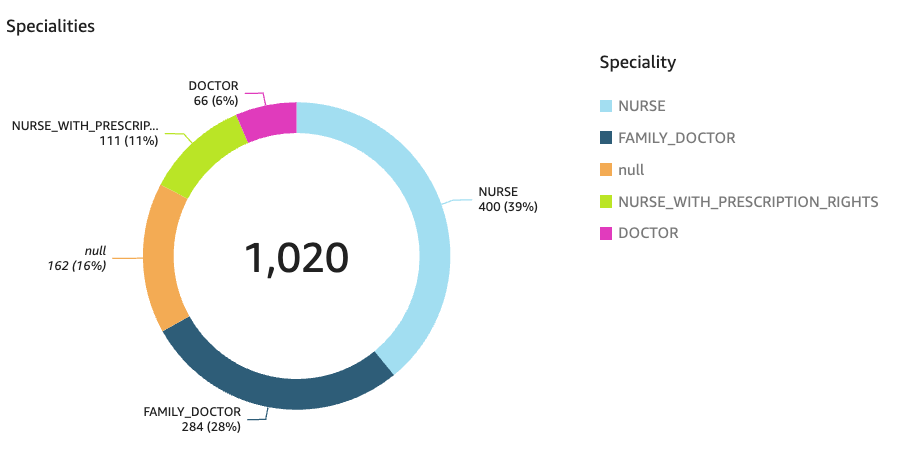


**Technical workflow:**

**Login and Authentication:** The platform uses multi-factor authentication (MFA) to ensure secure access, comparable to the level of security used for online banking. Patients and clinicians can log in using Estonia's widely adopted digital identification methods, such as Smart-ID, Mobile-ID, or ID card. This ensures that only verified users can access the system, protecting sensitive health data.

**Patient Panel Import and Monthly Updating:**

The platform includes an integration that allows clinics to securely import and update their patient panels, ensuring that only patients officially registered with the clinic can submit health queries. This process ensures that only registered patients can submit health queries, minimizing administrative errors and enhancing data security by verifying patient-clinic affiliations.

**Prescription Pathway:**
The prescription pathway allows patients to easily select the drug name or ingredient from a national drug database, reducing the need for free-text input. This structured approach minimizes spelling errors and inconsistencies in prescription requests, providing clinicians with standardized data that supports more reliable medication management and analysis.

**Condition-Based Pathways:**
The platform uses dynamic questionnaires that adjust based on patient responses to minimize the burden on the patient. The system also includes built-in red flag questions, which are sometimes overlooked during phone-based consultations. These questionnaires are designed based on European clinical guidelines, ensuring comprehensive and accurate data collection.

**Pre-Visit Anamnesis:**
The platform provides clinics with a structured, concise medical summary, or anamnesis, that clinicians can review and copy into the EHR. Clinicians also see how the summary was constructed by seeing the individual question-answer pairs, offering full transparency and control over the information being documented.

**Translation of E-Contacts:**
Certific integrates with a translation tool to facilitate the translation of e-contacts, ensuring smoother communication between patients and clinicians. In many countries, including Estonia, Electronic Health Records (EHR) are required by law to be documented in the national language. This can create a significant translation burden for clinicians when receiving patient communications in other languages.

**Patient Workflow:**

Certific’s platform is designed for ease of use, requiring no additional software installation or hardware investment. Both patients and clinicians access the platform conveniently via the same domain, [**www.perearst24.ee**](http://www.perearst24.ee) (translating to "General Practitioner 24"), using any internet-enabled device, such as smartphones, tablets, laptops, or PCs. This unified access point ensures flexibility and eliminates technological barriers for all users.

Patients log in using multi-factor authentication methods, such as Smart-ID, Mobile-ID, or ID cards, to ensure secure access.

The platform uses dynamic, condition-based pathways to guide patients through reporting their health concerns in a structured and concise manner (Figure 1). This approach minimizes the time required to input information while ensuring that physicians receive clinically relevant details. Patients select their main health concern from a list of available options and complete a short, adaptive questionnaire designed according to European medical guidelines (Figure 2). These pathways are tailored to gather all necessary information upfront, reducing the need for follow-up queries and enabling more efficient clinical decision-making.

Patients can also submit an e-contact on behalf of someone else, such as a child or an elderly family member. This feature is particularly beneficial for caregivers, as it allows them to manage health concerns for dependents securely through the platform.

Patients can upload images, files, and other supporting documents as part of their query. All files undergo virus scanning before being shared with clinicians, ensuring a secure exchange of health information and reducing the risk of cybersecurity threats.

Once a clinician has responded, patients receive an email notification, directing them to view the message on the platform. The platform maintains a complete history of all previous e-contacts, allowing patients to review past interactions at any time.

Patients may also respond to the clinician’s message if permitted. Certific employs a "clinician-controlled communication" feature, allowing healthcare providers to manage the communication flow, which will be further described in the next section.


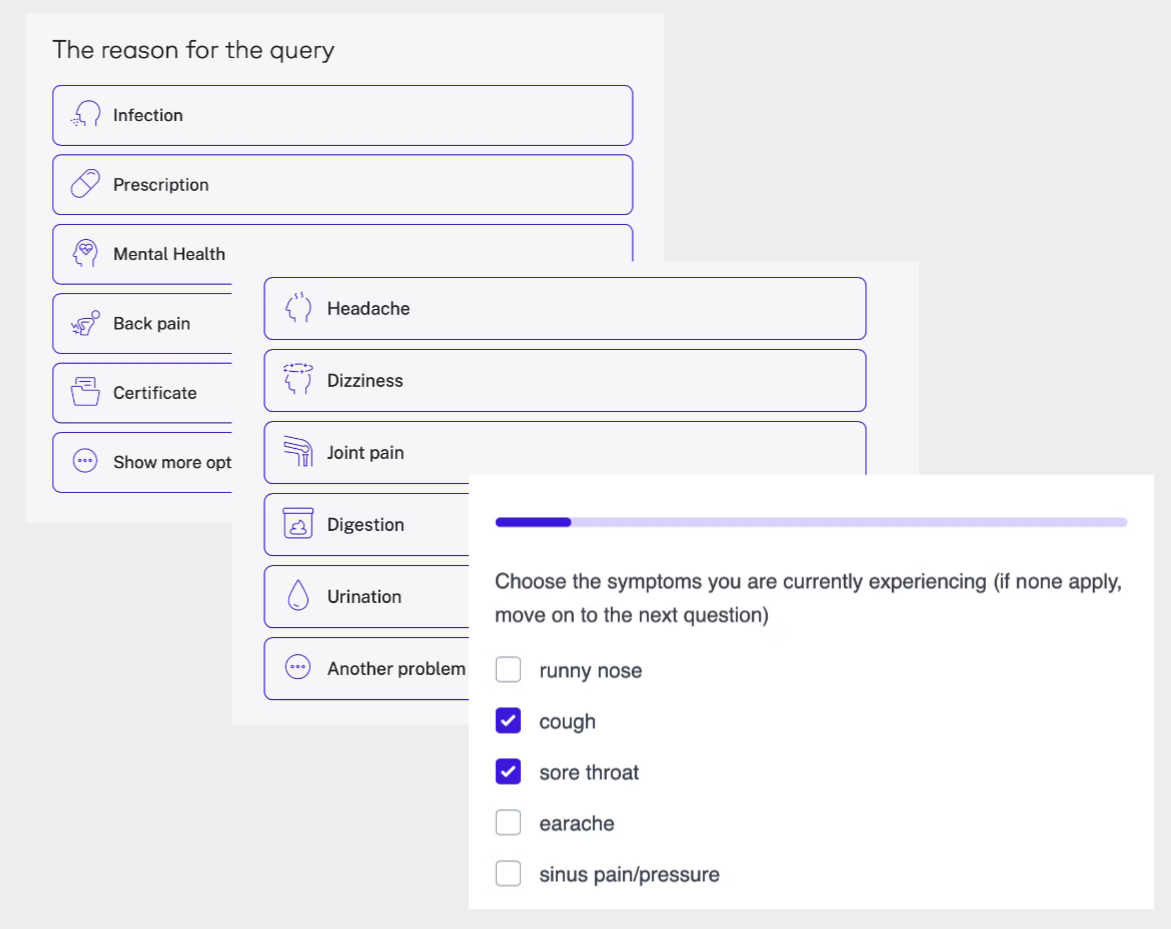


Figure 1. Condition-based pathways.


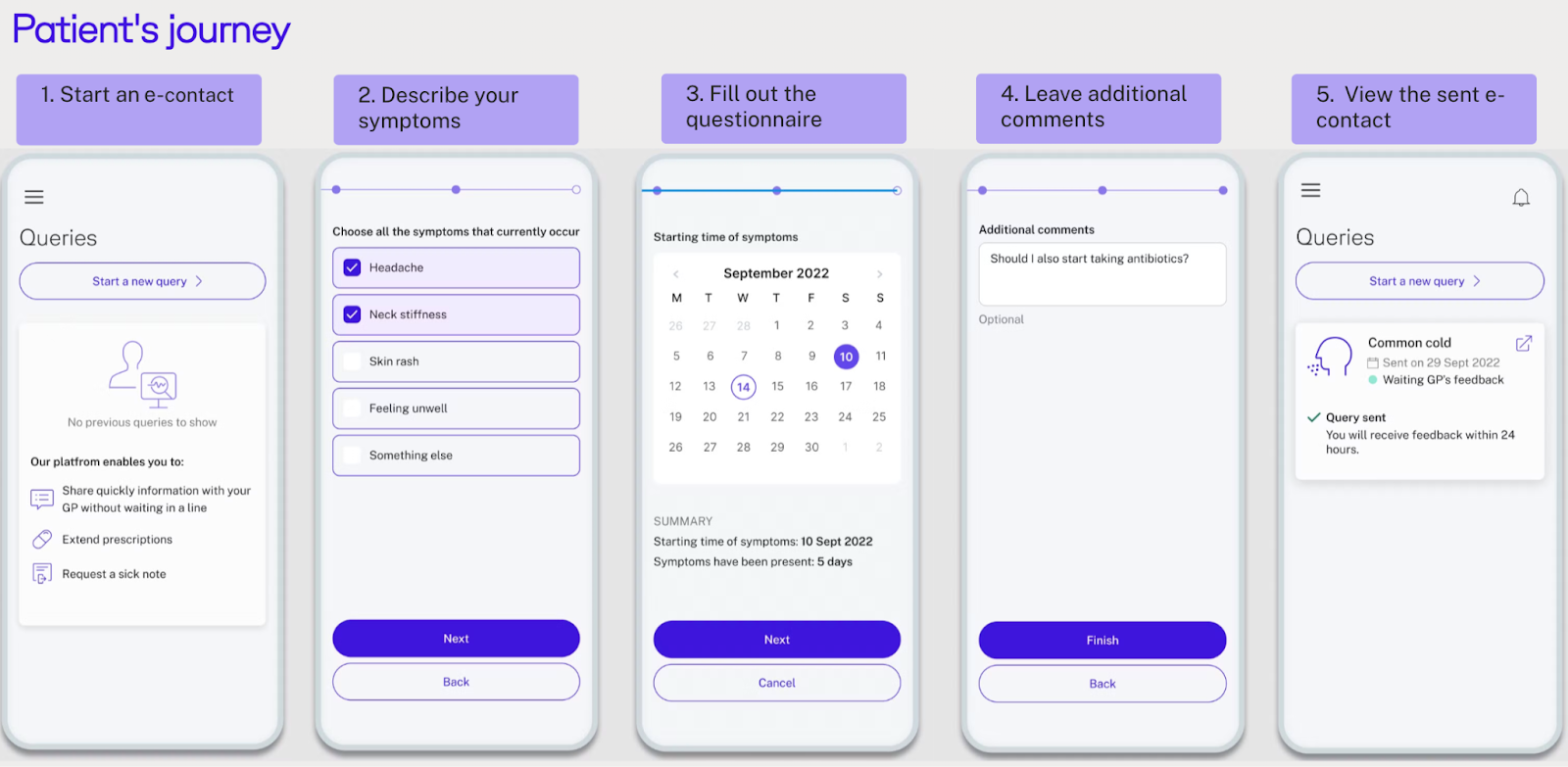


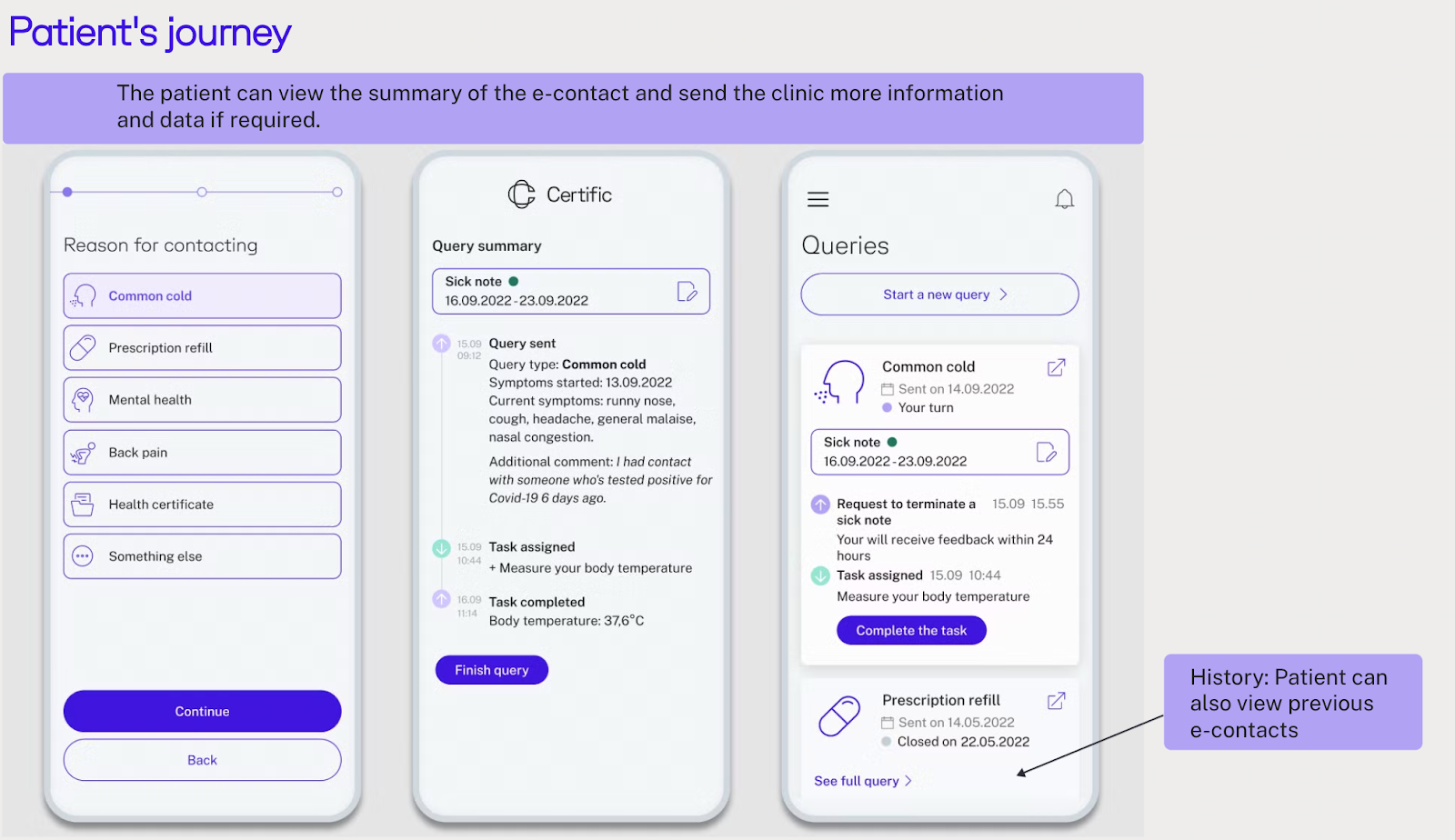


Figure 2. Example of the patient-facing symptom reporting pathway.

**Clinician workflow**

Dashboard Overview: Clinicians receive all incoming patient queries, known as e-contacts, directly on their dashboard (Figure 3). Each request is fully documented and ready for review, allowing clinicians to focus on patient care rather than administrative tasks. The dashboard provides various tools for managing e-contacts, including options to filter, sort, and search based on patient concerns, which aids in prioritizing cases and optimizing triage.


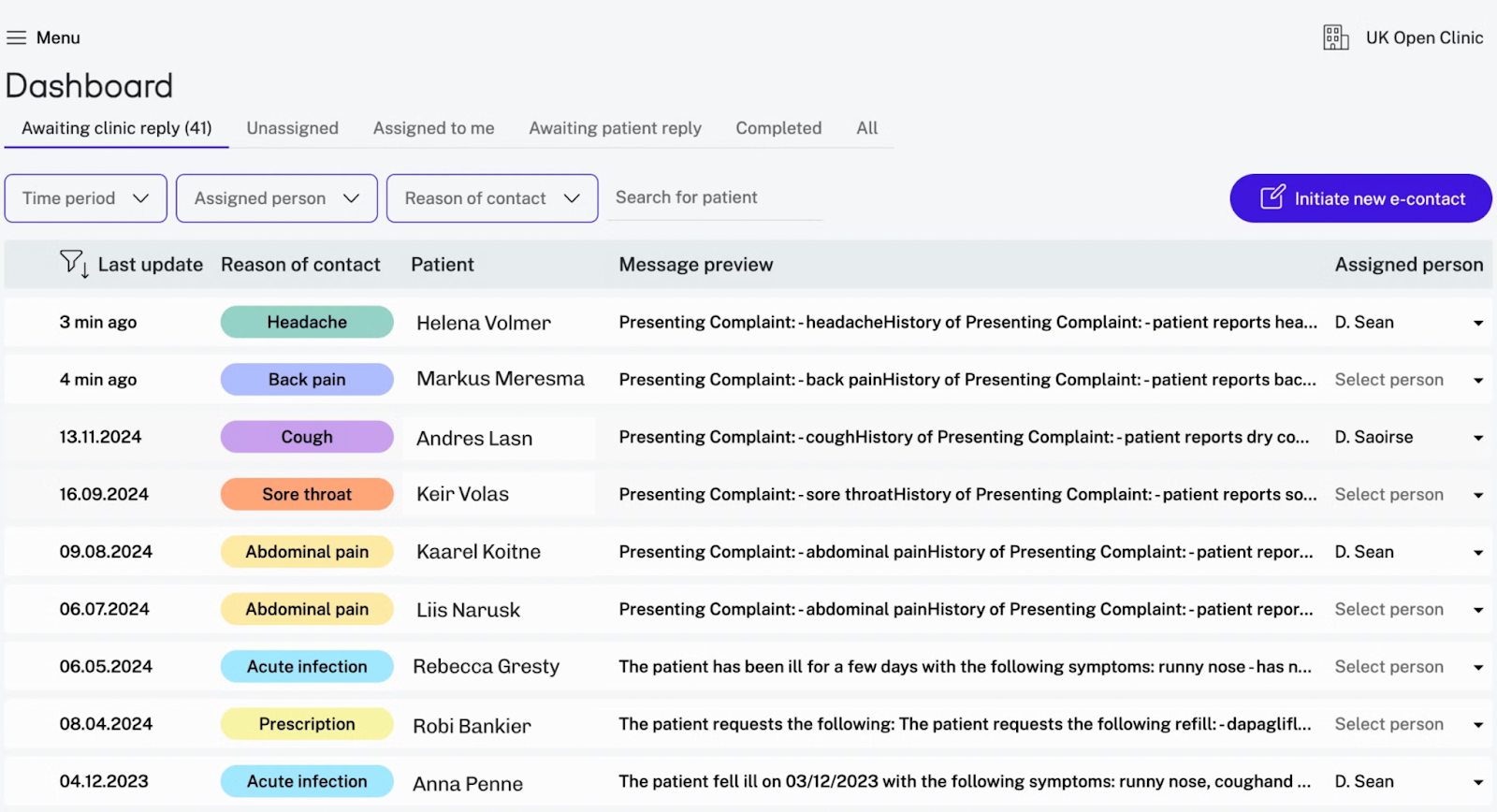


Figure 3. The dashboard view.

Detailed View of E-Contacts: In the detailed view of each e-contact, clinicians are presented with a comprehensive overview, including patient contact details, a concise medical anamnesis and all uploaded files, photos, and attachments, which are virus-scanned for security (Figure 4). Additionally, clinicians can view previous interactions between the patient and the clinic, enabling continuity of care.


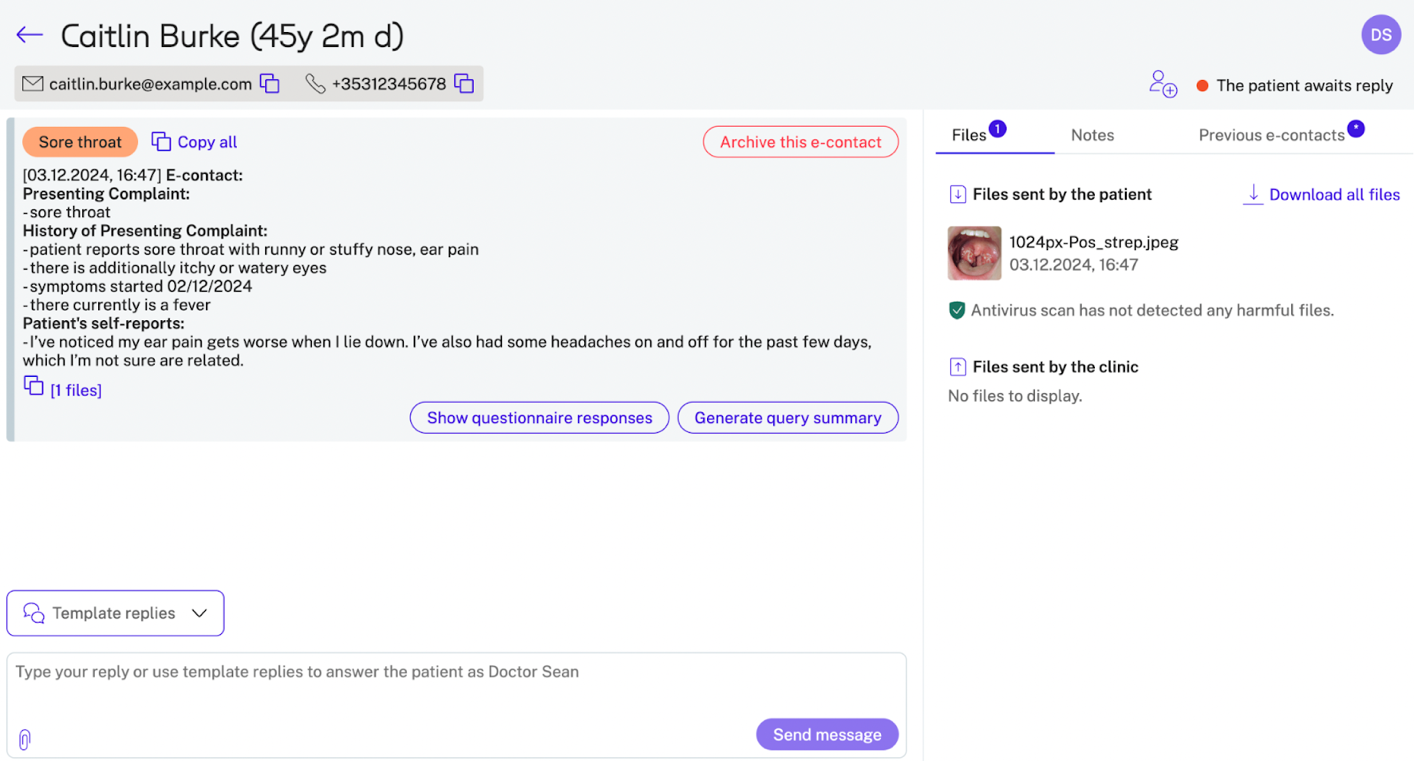


Figure 4. The detailed-view.

Pre-visit anamnesis: Certific’s platform drafts an anamnesis using patient input collected via dynamic, condition-based questionnaires. This summary can be reviewed and copied into the Electronic Health Record (EHR) with a simple copy-paste, significantly reducing the time spent on documentation. The platform balances structured data collection (multiple-choice inputs) with free-text fields, ensuring relevant information is captured while providing patients with the flexibility to add context. This process minimizes the need for follow-up questions and streamlines the documentation workflow.

Additional Standardized Questionnaires: If needed, clinicians can send patients additional standardized questionnaires to be completed before a follow-up appointment. This ensures that all relevant health information is gathered beforehand, optimizing the consultation process.

SMS Center: Clinicians have access to an SMS center, enabling them to send mass messages to patients. This feature is particularly useful for notifications such as vaccination reminders, screening invitations (e.g., colon cancer screening), or to encourage patients to adopt the platform during onboarding.

Triage and Categorization of E-Contacts: While the system does not automatically triage e-contacts, it categorizes each query with a title reflecting the patient’s main concern. This helps clinicians prioritize more urgent cases (e.g., acute infections or mental health issues) and manage their caseload efficiently. The platform offers additional capabilities not found in conventional communication tools such as email or WhatsApp, including task delegation, translation of e-contacts, template responses, and the ability to send clinical questionnaires.

Automation Tools for Efficiency: Certific includes several automation tools designed to increase clinical efficiency:

- Clinician-Controlled Communication: Clinicians can decide whether to close the conversation after responding or leave it open for further patient input, with the option to restrict when the patient can reply. This system helps reduce unnecessary communication and improves workflow efficiency.
- Clinician-Initiated Contact: Instead of chasing patients over the phone, clinicians can leave messages for patients, which they can access at their convenience in a secure, GDPR-compliant environment. This feature is often used to notify patients of test results or invite them to schedule appointments, such as vaccinations.
- Translation of E-Contacts: e-contacts can be translated with one click, addressing the challenge in countries like Estonia, where EHR documentation must be in the national language. This reduces the administrative burden on clinicians by automating the translation process, particularly for queries submitted in languages like English or Russian.
- Template Responses: Clinicians can create, use, and share template responses for common patient queries. Templates can be linked to specific problem types, reducing the time spent on repetitive communications and enhancing consistency in patient responses.
- SMS Functionality and Targeted Notifications: The platform includes an SMS center that enables clinics to send targeted notifications to patients. Clinicians can easily filter patients by criteria such as gender, age group, specific patient panels, or e-contact types (e.g., acute infection queries). As patients update their contact information upon logging in, the dataset remains current and of high quality, supporting effective and accurate communication with patient groups.

**Complaints made by typical patients**

Below is a summary of the demographics and presenting complaints made by patients, when they first adopted the Certific system in the primary care practices of this study.

**1. Sociodemographic data, on the types of patients using the website**

77% of e-contacts are submitted for the same user whose ID number was used to log into the platform (hereinafter the “query reporter”) while 23% of e-contacts were submitted for someone else (for example a child or other representative). The person for whom the e-contact was submitted is referred to as the “patient”.

*Gender*

Of the patients, 61% are female and 39% are male (Among query reporters, 70% are female and 30% are male, suggesting that females may be more likely to catering for the health needs of others).


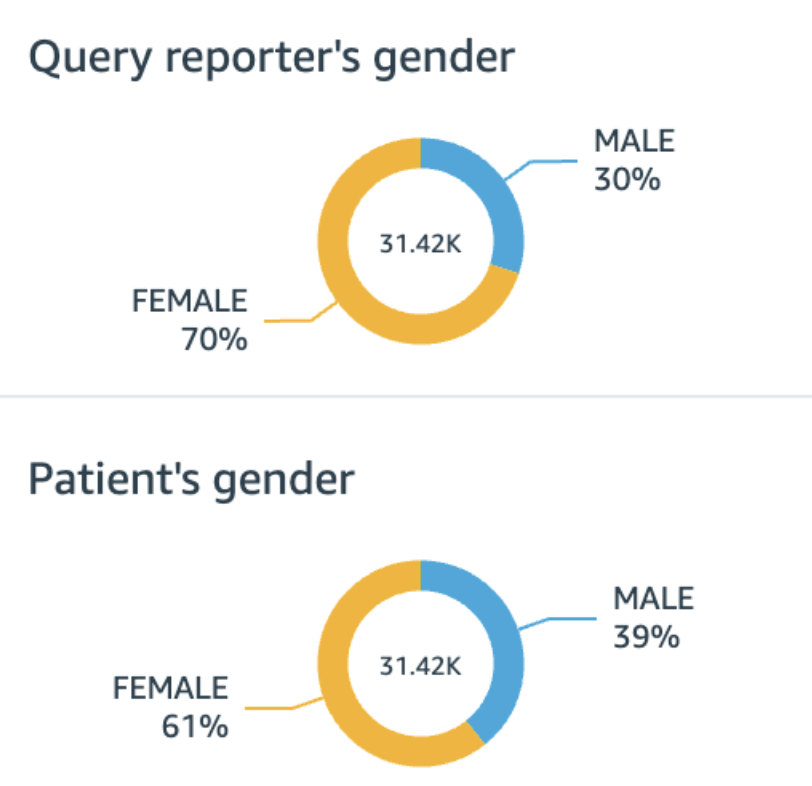


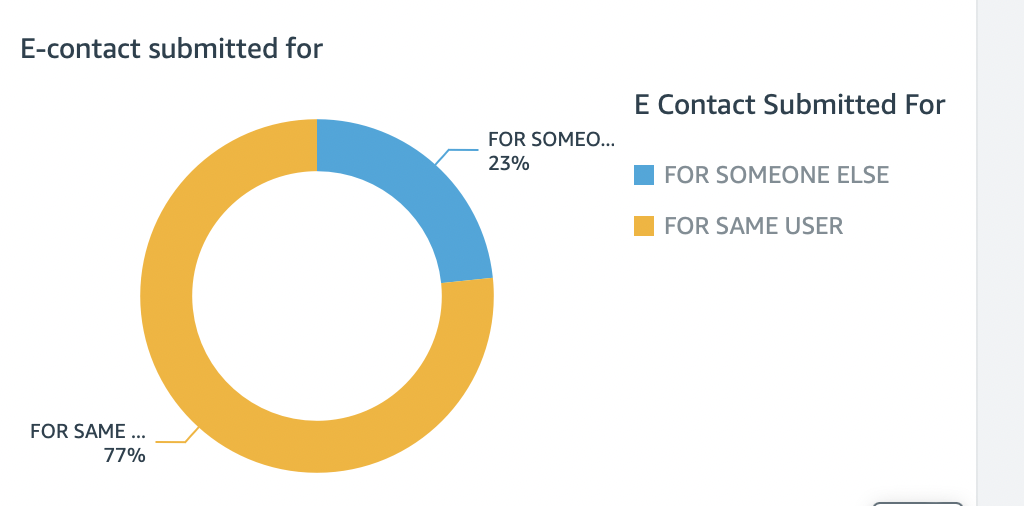


*Age*

Two histograms were constructed. One representing the age distribution of the Query Reporters and the other representing the age distribution of the Patients.

For the Query Reporters age distribution, the modal responder is in the early 30s, and the median age is 39.

For patients, the age distribution has a bimodal distribution. A pronounced peak is evident within the 0-10 age range, suggesting that a significant portion of the e-contacts initiated cater to this younger demographic. This suggests that the platform offers particular benefits for families with young children who perceive inputting information through e-channels as more convenient when compared to face-to-face care. A second peak is seen in the early 30s age bracket (e.g. the median patient age is 36).


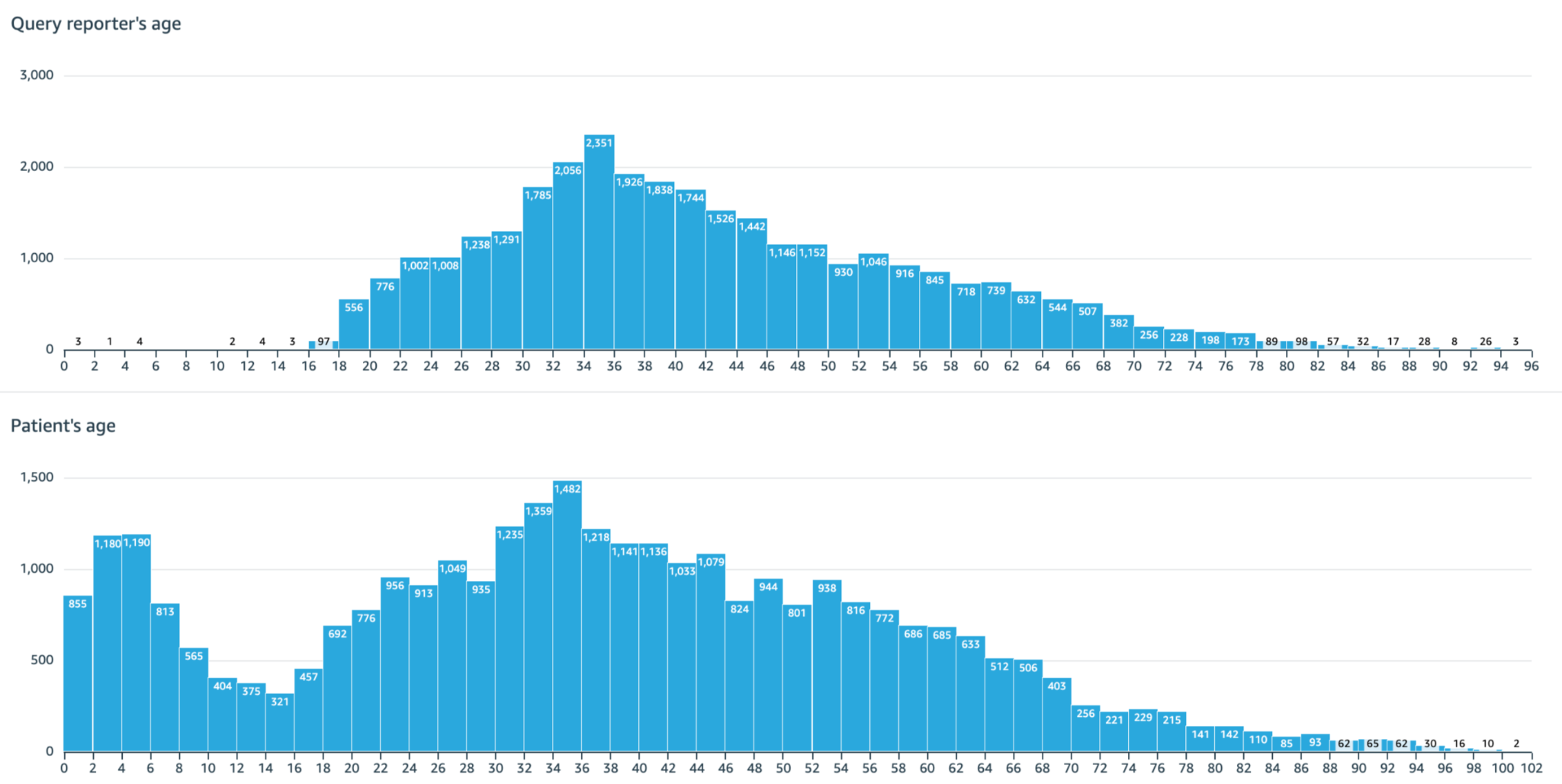
Query reporter = user who logged in their personal id/smart/m-id (the platform allows log in by the child's ID or their parents' ID).

Patient = for whom the e-contact was done (e.g can be also child).

**2. Direct patient feedback**

Methods

After a patient has completed an e-contact / e-consultation on Ceritifc's online-platform, each patient is asked to evaluate their experience using a five-point rating scale and provide unstructured feedback. This feedback covers perceived satisfaction with the service's speed, clarity, and ease of use/simplicity (screen shot below, in Estonian):


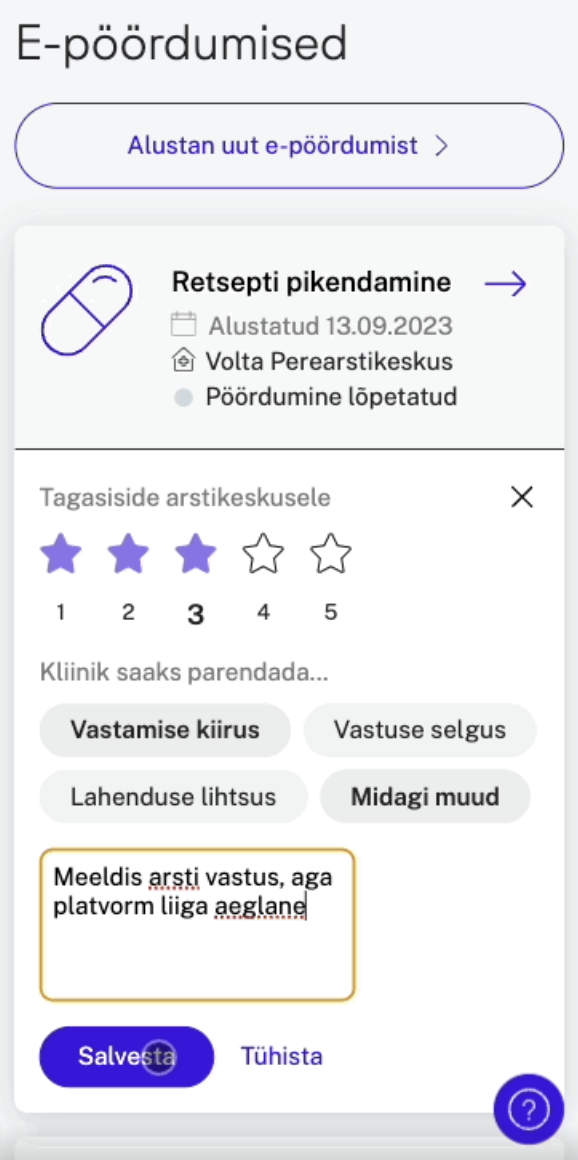


Results

From the date of the introduction of this feedback feature (23.02.2023) to when data was analyzed (13.09.2023) 27,360 patients who interacted with the platform during this time frame. Of these, 3241 individuals (11.8%) provided feedback (Table 1). While we cannot tell what satisfaction was like among the 88% of nonresponders, among these responders the average patient feedback rating was 4.84 on a five-point scale.

Table 1


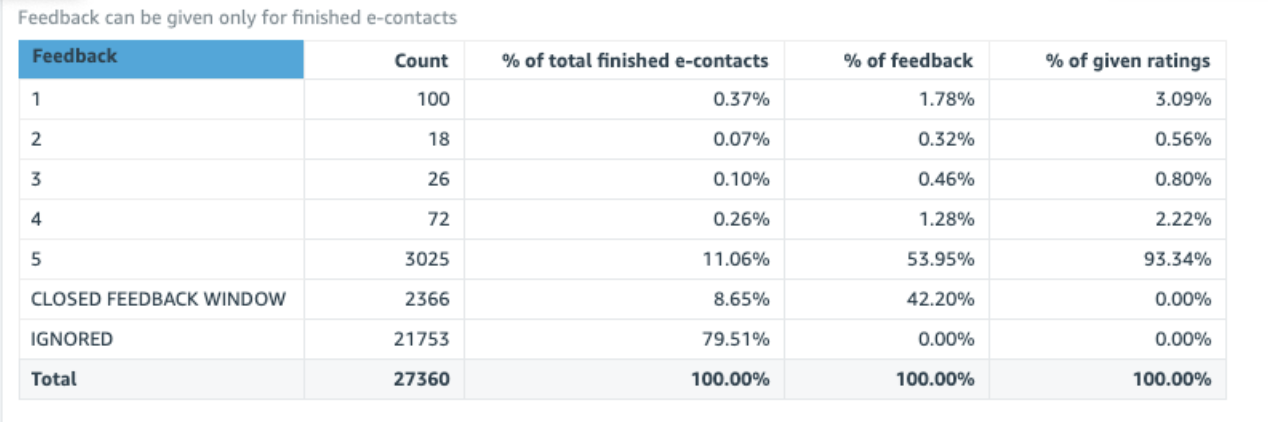


**3. Type of clinical presentation**

Preliminary data is as follows. Between 14.12.2022 – 20.09.2023, 33,138 patients initiated an electronic health query (e-query), across 41 primary care practices.

(Of these, 15,481 (65%) queries were marked as resolved without requiring further e-communications. However, some of these patients may have received a phone call from their practice nurse. Accordingly, we cannot quantity the exact number whose clinical problem was resolved after a single episode of data entry. However, this is likely to be around half of the user base.)

We analyzed what type of problem the patient reported. Its distribution is as follows:

**
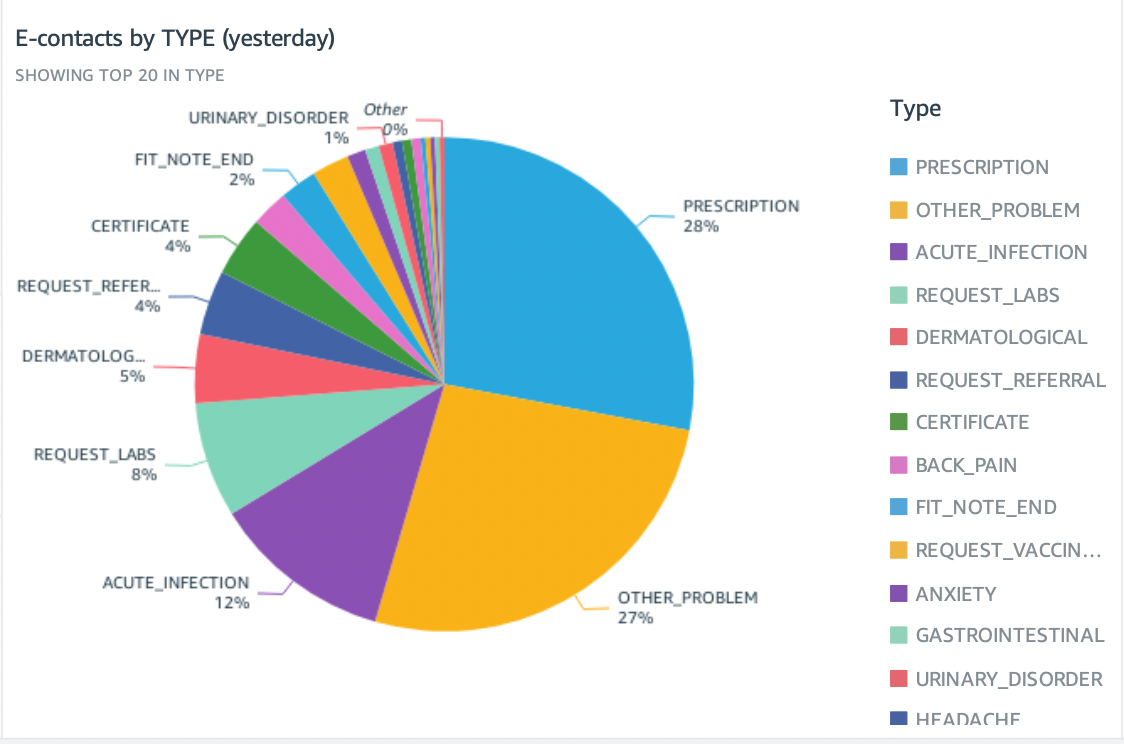
**

For 27%, the type was not specified. Among those queries where it was specified, in most cases (63%) the e-query was about a preexisting disease or health state that required electronic follow-up (renewing a prescription, a blood test, a referral or a certificate). Of the newly presenting symptoms, these were mainly infections (16%), skin complaints (7%), or urinary symptoms (1%). Altogether, suggests that the e-query system may be particularly suitable for patients with a known healthcare condition that may be chronic in nature, and sometimes requiring just clerical or light-touch chronic disease management options.
