# Supplementary material for: Acceptability of Guided Symptom Entry and Asynchronous Clinical Communication Software Among Primary Care Staff: Qualitative Study
Source: JMIR Form Res. 2025 Jul 16;9:e59620. doi: 10.2196/59620 (PMC12286587; doi:10.2196/59620)
Supplement: Multimedia Appendix 2 [file formative-v9-e59620-s002.docx]

Example of themes, categories and codes derived from inductive coding.

| **Theme** | **Category** | **Code** |
| --- | --- | --- |
| Advance of remote consulting | Begun in 2010s | Initially phone and e-mail |
|  |  | New normal since COVID |
|  |  | Practice no longer has walk-ins, pre-agreed appointments only |
|  |  | Have had to adjust working practices |
|  |  | More change for nurses than doctors |
| “Patients are changing” | More impolite | 24-hour availability impression |
|  |  | Lack of perceived boundaries |
|  | More helpless | Come with same issue several times |
|  |  | Do not know how to describe symptoms |
|  |  | Afraid of making a phone call |
|  |  | Even young people do not know the basics of how to take care of self, child |
| Patients are varied | Digital literacy varies | Among older people there are digitally literate people |
|  |  | Among younger, digitally challenged |
|  |  | Language can be a barrier |
|  |  | Some refuse to accept “cookies” on their computer. |
|  | Expression of pain or trouble varies | Some pt overstate in writing |
|  |  | Some understate in writing |
|  | Perception of inappropriate queries | Too casual, almost frivolous query |
|  |  | Refusing responsibility for simple life tasks (e.g asking same information a number of times) |
|  |  | Expressing too much emotion irrelevant to query |
|  |  | Pt believed to be seeking company |
|  | Expresses a wish to restrict inflow of pt info | Need “gatekeeper to the gatekeeper” |
| E-mail | Ease of documentation | Allows copy and paste |
|  |  | Written record may make things easier |
|  | Asynchronicity | Patients can access in their own time |
|  |  | Patients may abuse by writing frivolous e-mails whenever |
|  |  | Allows provider to take time to think and respond |
|  | Access | Most patients now proficient in using |
|  | Unpredictable content | Too little information relevant to diagnosis |
|  |  | E-mail too long, information irrelevant |
|  |  | Anonymous pt e-mail, no id |
|  |  | Impolite content |
|  |  | Non-medical content |
|  |  | Content perceived to be frivolous, not in good faith |
|  |  | “Chat-like” one-line opening or invitation for a conversation |
|  | Time-consuming clarification | Provider writes for clarification |
|  |  | Insufficient response from patient |
|  |  | Following up with a call |
|  |  | Relevant info in several e-mail |
|  |  | Compiling information for coherent documentation is difficult, requires concentration |
|  | Workflow constantly interrupted | Cannot complete a query/triage due to missing information |
|  |  | Phone call disrupts working on another e-mail query |
|  | Getting documentation right is important and stressful | Hard to find info from different e-mails for summarizing |
| Phone | Synchronous communication enables definitive solution | When agreeing on appointment times |
|  |  | Enables definitively solve certain things |
|  |  | Enables asking additional information |
|  |  | Enables checking for understanding. |
|  | Enables hearing patient voice | Adds weight to diagnosis hypothesis |
|  |  | Understanding of urgency - how bad is it |
| Certific | Summary of symptoms | Summary makes documentation easier |
|  |  | Summary is comprehensive, satisfies |
|  |  | Can edit summary |
|  |  | Can copy straight to EHR |
|  |  | Eases cognitive burden-don´t have to compile info |
|  | ID-based login | Resolves pt identity issue |
|  |  |  |
