# Supplementary material for: Acceptability of Guided Symptom Entry and Asynchronous Clinical Communication Software Among Primary Care Staff: Qualitative Study
Source: JMIR Form Res. 2025 Jul 16;9:e59620. doi: 10.2196/59620 (PMC12286587; doi:10.2196/59620)
Supplement: Multimedia Appendix 3 [file formative-v9-e59620-s003.docx]

**Appendix 3. Sample description.**

| Practice | Location | Approximate # of patients served | Characteristics of population served | Role | Gender | Age |
| --- | --- | --- | --- | --- | --- | --- |
| Practice 1 | Other large city | 1900 | Homogenous | GP1 | F | Early 60s |
|  |  |  |  | Nurse 1.1 | F | 50s |
|  |  |  |  | Nurse 1.2 | F | Mid- 50s |
| Practice 2 | Suburb near capital | 10 000 | Multi-ethnic | GP 2 | F | Early 50s |
|  |  |  |  | Nurse 2 | F | Early 40s |
| Practice 3 | Capital | 10 000 | Multi-ethnic | GP 3 | F | Late 40s |
| Practice 4 | Suburb near capital | 8000 | Multi-ethnic | GP 4 | F | Early 60s |
|  |  |  |  | Nurse 4 | F | Early 30s |
| Practice 5 | Suburb near capital | 7000 | Homogenous | GP5 | F | Early 30s |
| Practice 6 | Capital | 3500 | multi-ethnic | GP6 | F | Early 60s |
|  |  |  |  | Nurse 6 | F | Late 50s |
| Practice 7 | Other large city | 1800 | homogenous | Nurse 7.1 | F | Mid-30s |
|  |  |  |  | Nurse 7.2 | F |  |
| Practice 8 | Other large city | 2000 | homogenous | Nurse 8 | F |  |
